# Omega-3 PUFA Loaded in Resveratrol-Based Solid Lipid Nanoparticles: Physicochemical Properties and Antineoplastic Activities in Human Colorectal Cancer Cells In Vitro

**DOI:** 10.3390/ijms19020586

**Published:** 2018-02-16

**Authors:** Simona Serini, Roberta Cassano, Paola Antonia Corsetto, Angela Maria Rizzo, Gabriella Calviello, Sonia Trombino

**Affiliations:** 1 Institute of General Pathology, School of Medicine, Università Cattolica del Sacro Cuore, Largo F. Vito, 1, 00168 Roma, Italy; simona.serini@unicatt.it; 2 Department of Pharmacy, Health and Nutritional Sciences, Università della Calabria, Via Pietro Bucci, 87036 Arcavacata di Rende, Cosenza, Italy; roberta.cassano@unical.it (R.C.); sonia.trombino@unical.it (S.T.); 3 Department of Pharmacological and Biomolecular Sciences, Università degli Studi di Milano, Via D. Trentacoste, 2, 20134 Milan, Italy; paola.corsetto@unimi.it (P.A.C.); angelamaria.rizzo@unimi.it (A.M.R.)

**Keywords:** colon cancer cells, delivery, encapsulation, nanoparticles, omega-3, resveratrol

## Abstract

New strategies are being investigated to ameliorate the efficacy and reduce the toxicity of the drugs currently used in colorectal cancer (CRC), one of the most common malignancies in the Western world. Data have been accumulated demonstrating that the antineoplastic therapies with either conventional or single-targeted drugs could take advantage from a combined treatment with omega-3 polyunsaturated fatty acids (omega-3 PUFA). These nutrients, shown to be safe at the dosage generally used in human trials, are able to modulate molecules involved in colon cancer cell growth and survival. They have also the potential to act against inflammation, which plays a critical role in CRC development, and to increase the anti-cancer immune response. In the present study, omega-3 PUFA were encapsulated in solid lipid nanoparticles (SLN) having a lipid matrix containing resveratrol esterified to stearic acid. Our aim was to increase the efficiency of the incorporation of these fatty acids into the cells and prevent their peroxidation and degradation. The Resveratrol-based SLN were characterized and investigated for their antioxidant activity. It was observed that the encapsulation of omega-3 PUFA into the SLN enhanced significantly their incorporation in human HT-29 CRC cells in vitro, and their growth inhibitory effects in these cancer cells, mainly by reducing cell proliferation.

## 1. Introduction

Colorectal cancer (CRC) is the third most common cancer worldwide, and environmental, genetic and epigenetic factors are involved in its carcinogenesis [1,2,3]. Currently, besides surgical removal, both conventional (chemotherapy, and radiotherapy) and innovative approaches (personalized single-targeted therapies) are used for the cure of this cancer [4,5,6,7], which, in case of early diagnosis, represents one of the most curable ones. However, these therapies often induce significant side-effects [8,9], and their long-term effectiveness may be challenged by acquired resistance [10]. Additional problems for single-targeted therapies are the high costs and progressive alterations of additional molecular targets during CRC progression.

One proposed strategy to efficiently enhance the effectiveness of these existing treatments is to combine them with increased intakes of cheap dietary components showing “multitargeted” antineoplastic activity, such as long-chain omega-3 polyunsaturated fatty acids (omega-3 PUFA). Most of the in vitro and in vivo studies [11,12] have demonstrated the safety and protective actions of these dietary fatty acids (FA), especially against the development and progression of CRC, and the most common hormone-related cancer (breast and prostate cancer). Different markers and signaling pathways involved in the development and progression of these cancers have been shown to be negatively regulated by omega-3 PUFA, including those involved in apoptosis and cell proliferation [13,14,15]. In particular, plenty of studies performed both in vitro and in vivo in different kinds of cancer have demonstrated the ability of these fatty acids to inhibit cell proliferation and induce apoptosis [16,17,18,19,20,21,22,23,24] These fatty acids have been However, some positive associations were recently found between the incidence/progression of human prostate cancer and high levels of intake or high concentrations of these fatty acids (FA) in blood or prostate tissue [25]. Even though other factors (such as individual genetic profiles or carcinogenic contaminants present in the dietary sources of these FA) may be more directly involved in prostate carcinogenesis [11], the alarming positive associations prompted to suggest that extremely high levels of omega-3 PUFA intake should be avoided. Actually, plenty of outcomes support the hypothesis that omega-3 PUFA may induce either beneficial or deleterious effects depending on the levels at which they are administered [26]. The potential dangerousness of extremely high levels of omega-3 PUFA has been generally related to their unsaturated structure, which makes them highly susceptible to oxidation. This may result into reduced bioactivity, and cell oxidative stress, that may induce toxic and carcinogenic effects [11]. This possibility cannot be ignored, and thus, for a potential application of these FA in the adjuvant therapy of CRC, it would be important to supply colonic mucosa cells with relatively moderate concentrations of these FA, that, however, should still result able to inhibit CRC cell growth.

One possibility to reduce the doses of omega-3 PUFA could be to protect them from environmental conditions and chemical changes during their administration and absorption, with the aim to enhance their stability and, consequently, also their antineoplastic efficiency. One interesting approach developed in recent years to obtain these results for a number of bioactive dietary factors is their encapsulation in nano-delivery systems, such as solid lipid nanoparticles (SLN). These nanoparticles have also been shown to provide more surface, increase solubility, improve the controlled release of drugs/nutraceutics, as well as their active targeting [27].

In the present work, we hypothesized that omega-3 PUFA encapsulation in SLN with a lipid matrix constituted by stearic acid (C_18_H_36_O_2_) esterified to the dietary polyphenolic phytochemical resveratrol (RV, R3,5,4′-trihydroxy-*trans*-stilbene), besides imparting further hydrophobicity to these omega-3 PUFA and potentially improving their delivery to CRC cells, could also protect omega-3 PUFA from oxidation and degradation, and, thus, enhance their antineoplastic activity. Moreover, RV is known to prevent CRC development and progression with a “multitargeted” mode of action [28], which, for many aspects, is similar to that exhibited by omega-3 PUFA, and the outcomes of two clinical trials also revealed the anticarcinogenic potential of oral RV administrations in normal and malignant colorectal tissues of patients [29,30]. We developed and characterized these RV-based solid lioid nanoparticles (RV-SLN), and encapsulated in them either docosahexaenoic acid (DHA, 22:6ω-3) or its metabolic precursor, α-linolenic acid (LNA, 18:3ω-3). We characterized the newly developed RV-SLN by evaluating their physicochemical properties and antioxidant activity. Moreover, we investigated whether the encapsulation of LNA or DHA into the RV-SLN could modify the incorporation of these omega-3 PUFA in human HT-29 CRC cells in vitro, and enhance their inhibitory effect on the growth of these cancer cells.

## 2. Results

### 2.1. Preparation and Physicochemical Properties of Resveratrol-Stearate-Solid Lipid Nanoparticles (RV-SLN)

**Scheme 1:**
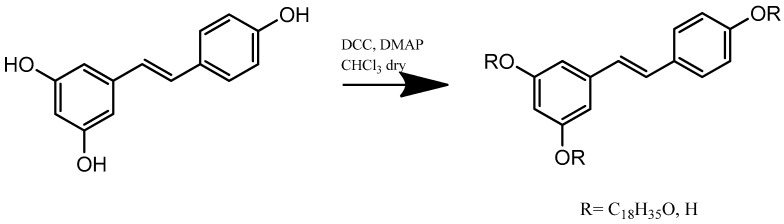
Synthetic route for resveratrol-stearate preparation.

In this work, a new lipophilic and antioxidant compound was obtained by an esterification reaction between RV and stearic acid (Scheme 1). This represented the lipid matrix for the synthesis of a new type of SLN. We confirmed by spectroscopy FT-IR(KBr) v (cm^−1^) the formation of the product: 3326 (OH), 3127, 3033 (CH), 2929, 2850 (CH), 1762 (C=O) 1382 (OH).^1^H-NMR (CDCl_3_: δ 0.86 (3H, t), 1.15–1.57 (30H, m), 2.41 (2H, t), 6.23 (1H, dd), 6.83 (2H, m), 7.18–7.31 (6H, m). Yield: 75%.

**Figure 1:**
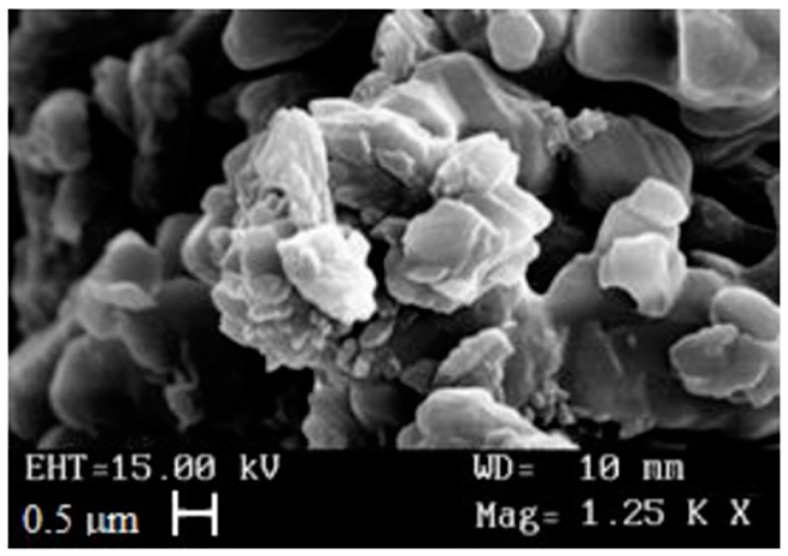
Photomicrograph of RV-SLN.

The RV-SLN, either empty or loaded with LNA or DHA, were prepared using the microemulsion technique [31,32,33,34,35]. The average nanoparticle diameter and their polydispersity index (PI), shown in Table 1, were determined through light scattering. The SLN dimensions were confirmed through scanning electron microscopy (SEM) that also highlighted the nanoparticles spherical shape (Figure 1). Both the average diameter and the PI of LNA-RV-SLN were lower than those obtained for the DHA-RV-SLN. In any case, the PI values obtained for the different nanoparticles analyzed (empty, or containing LNA or DHA) were quite low (≤220) and indicative of a good homogeneity in the distribution of particle sizes. Moreover, the SLN were characterized through UV-Vis spectrophotometer to evaluate the percentage of LNA and DHA encapsulation. The encapsulation efficiency (EE) obtained for LNA and DHA was of 77% and 100%, respectively, demonstrating that both the PUFA (and especially DHA) had a high chemical affinity for the ester composing the nanoparticles.

### 2.2. Antioxidant Ability of RV-SLN

**Figure 2:**
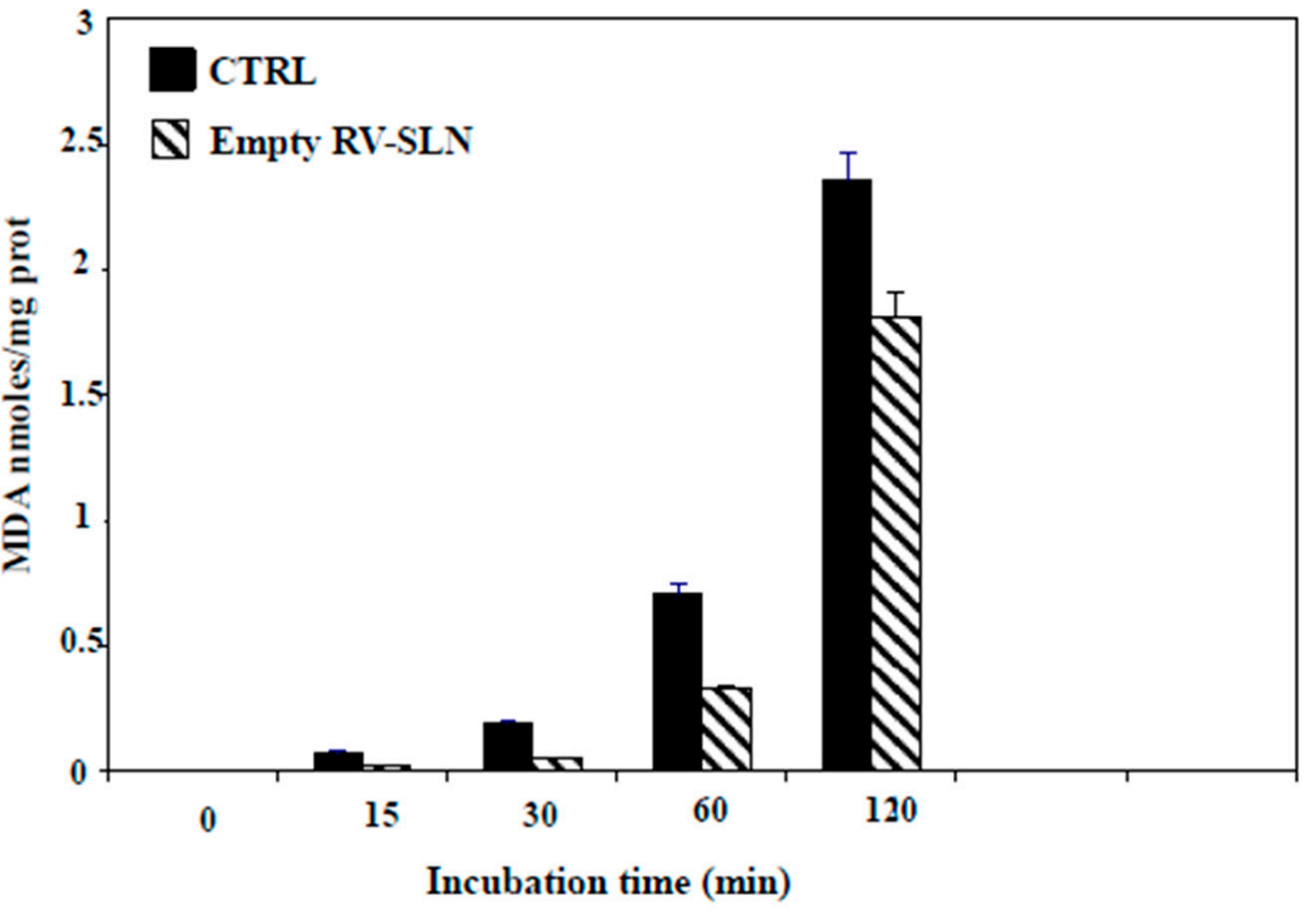
MDA (malondialdehyde) production in microsomal membranes incubated in the absence or in the presence RV-SLN. The microsomal membranes (1 mL, 0.5 mg proteins) were incubated with 0.25 × 10^−3^ M *t*-BOOH at 37 °C under air in the dark either in the absence (control) or in the presence of 0.8 µM empty RV-SLN for increasing periods of time (0–120 min). MDA production is expressed as nmoles/mg prot. Data are expressed as means ± standard deviation (SD) of three different experiments. Overall *p* < 0.05 (two-way Analysis of Variance (ANOVA) test, followed by Bonferroni’s post-test).

We next evaluated the antioxidant ability of the RV-SLN. Rat-liver microsomal membranes were incubated in the presence or in the absence of RV-SLN and subject to lipid peroxidation induced in vitro by *tert*-BOOH (*t*-BOOH). The results showed that the RV-SLN acted as efficient antioxidants, being able to protect the microsomal membranes from *t*-BOOH-induced lipid peroxidation. Particularly, RV-SLN were able to significantly inhibit (by about 25% at 2 h) the production of malondialdehyde (MDA) observed in microsomal membranes incubated in the absence of RV-SLN (Figure 2).

### 2.3. Omega-3 Incorporation in HT-29 Colorectal Cancer Cells (CRC)

**Figure 3:**
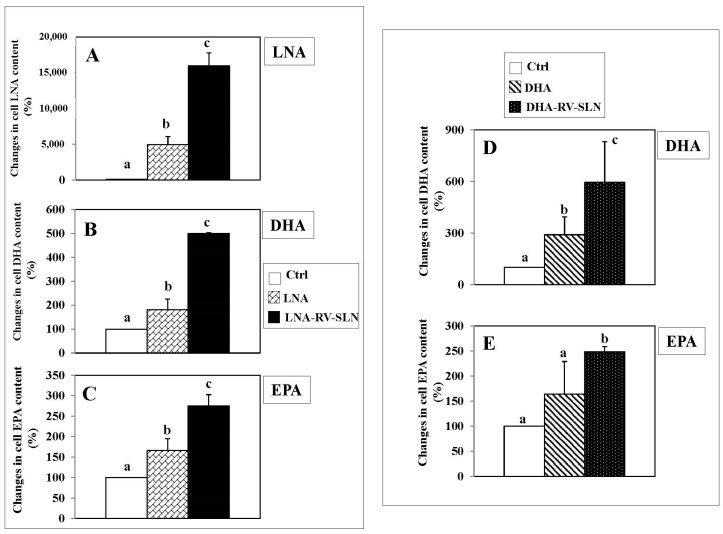
Changes in LNA, DHA and EPA content in HT-29 cells treated with either LNA or DHA administered in the free or encapsulated form. (**A**–**C**) cells were treated with either 50 µM LNA or 50 µM LNA-RV-SLN- for 24 h; (**D**–**E**) cells were treated with either 50 µM DHA or 50 µM DHA-RV-SLN for 24 h. Values are the means ± SD of three different measurements. Values not sharing the same superscript are significantly different (*p* < 0.05, One-way ANOVA, followed by Tukey’s test).

We evaluated by gas-chromatography if the encapsulation of DHA and LNA in RV-SLN could enhance the incorporation of these FA in HT-29 cells. We found that the encapsulation of LNA increased dramatically and significantly its incorporation in HT-29 cells after 24 h incubation (LNA-RV-SLN vs. free LNA: increase in cell content, 222.7%, *p* < 0.02) (Figure 3A). Moreover, when encapsulated in RV-SLN, LNA induced also a more conspicuous incorporation of its metabolic products EPA and DHA (LNA-RV-SLN vs. free LNA: DHA content increase, 277.2%, *p* < 0.009; EPA cell content increase, 165.7%, *p* < 0.03). Similarly, we observed a higher increase in the incorporation of DHA at 24 h when it was administered encapsulated (DHA-RV-SLN vs. free DHA: increase in DHA cell content: 204.8%) (Figure 3B). DHA was able to induce also an increase in the content of EPA, originated by DHA retroconversion. Of note, the cellular increase in EPA content reached the significance only when DHA was administered encapsulated in RV-SLN (DHA-RV-SLN vs. CTRL: increase in EPA cell content: 151.3%).

### 2.4. Effects of RV-SLN on Tumor Cell Growth

**Figure 4:**
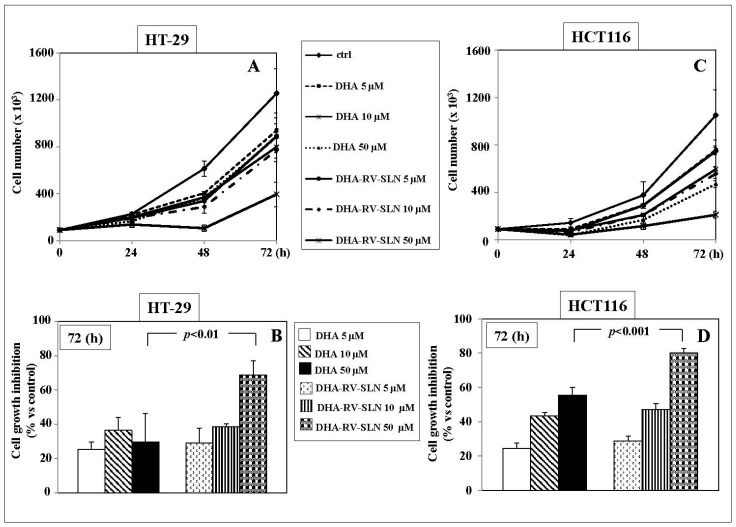
Effect of free DHA or DHA encapsulated in RV-SLN on the growth of HT-29 and HCT116 CRC cells. (**A**,**C**) Time-dependent (0–72 h) effect of increasing concentrations (5–50 µM) of free DHA or DHA-RV-SLN on the growth of HT-29 and HCT116 CRC cells. Data are the means ± SD of three different experiments; (**B**,**D**). Effect of a 72 h-treatment with free DHA or DHA-RV-SLN (50 µM) on the growth of HT-29 and HCT116 CRC cells. Data are the means ± SD of three different experiments. The significance value shown has been calculated by unpaired *t*-test.

The effects of the treatment with increasing concentrations of free DHA or DHA-RV-SLN (5–50 µM) on the growth of the human HT-29 and HCT116 adenocarcinoma cell lines are shown in Figure 4. Both free DHA and DHA-RV-SLN induced a time-dependent inhibition of both the CRC cell growth at all the concentrations tested (Figure 4A,B). Starting from 48 h, and even more markedly after 72 h, 50 µM DHA-RV-SLN induced in both the cell lines a significantly higher cell growth inhibition (*p* < 0.01 and *p* < 0.001 in HT29 and HCT116 cells, respectively) than free DHA used at the same concentration (inhibition vs. control: HT-29, DHA 50 µM, 29.7%; 50 µM DHA-RV-SLN, 68.6%. HCT116: DHA 50 µM: 55.3%; 50 µM DHA-RV-SLN: 80%) (Figure 4A,B).

**Figure 5:**
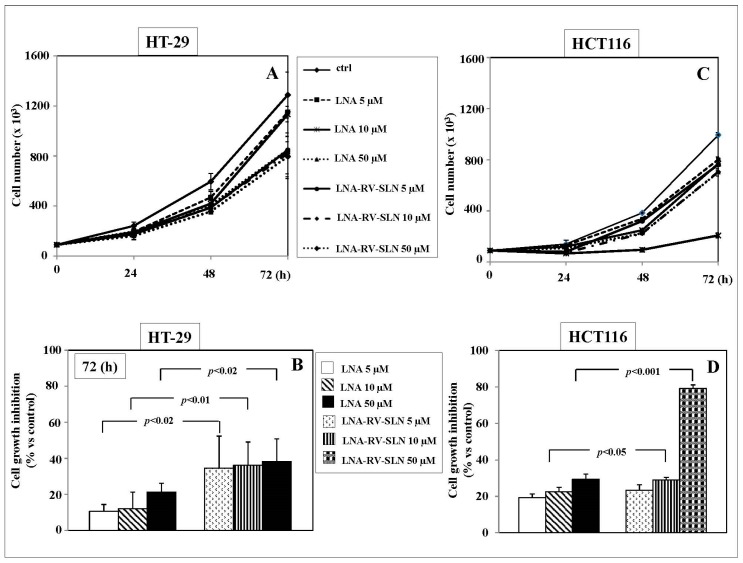
Effect of free LNA or LNA encapsulated in RV-SLN on the growth of HT-29 and HCT116 CRC cells. (**A**,**C**) Time-dependent (0–72 h) effect of increasing concentrations (5–50 µM) of free LNA or LNA-RV-SLN on the growth of HT-29 and HCT116 CRC cells. Data are the means ± SD of three different experiments; (**B**,**D**). Effect of a 72h-treatment with free LNA or LNA-RV-SLN at the concentration of 50 µM on the growth of HT-29 and HCT116 CRC cells. Data are the means ± SD of three different experiments. The significance values shown have been calculated by unpaired *t*-test.

Moreover, we aimed to evaluate if also LNA, the essential metabolic precursor of the longer-chain and more bioactive omega-3 PUFA EPA and DHA, could inhibit the growth of the human HT-29 and HCT116 adenocarcinoma cell lines (Figure 5A,B). Also LNA was able to significantly inhibit the growth of HT-29 and HCT116 cells starting from 48 h treatment (Figure 5A,B). The effect was even more remarkable following the 72 h treatment (Figure 5A,B). Interestingly, in HT-29 cells LNA delivered to cells by RV-SLN resulted significantly (*p* < 0.02) more efficient than free LNA in inhibiting tumor cell growth at all the concentrations analyzed (HT-29 cell growth inhibition vs. control: 5 µM LNA, 10.7%; 5 µM LNA-RV-SLN, 34.6%; 10 µM LNA, 12%; 10 µM LNA-RV-SLN, 36.3%; 50 µM LNA, 2.3%; 50 µM LNA-RV-SLN, 38.2%) (Figure 5B). In HCT116 cells, LNA delivered to cells encapsulated in RV-SLN was found to be significantly more efficient (*p* < 0.05 and *p* < 0.001, respectively) than free LNA at the concentrations of 10 and 50 µM (HCT116 cell growth inhibition vs. control: 5 µM LNA, 19.3%; 5 µM LNA-RV-SLN, 23.3%; 10 µM LNA, 22.5%; 10 µM LNA-RV-SLN, 29%; 50 µM LNA, 29.4%; 50 µM LNA-RV-SLN, 79.1%) (Figure 5D).

### 2.5. Effects of RV-SLN on Apoptosis Induction

**Figure 6:**
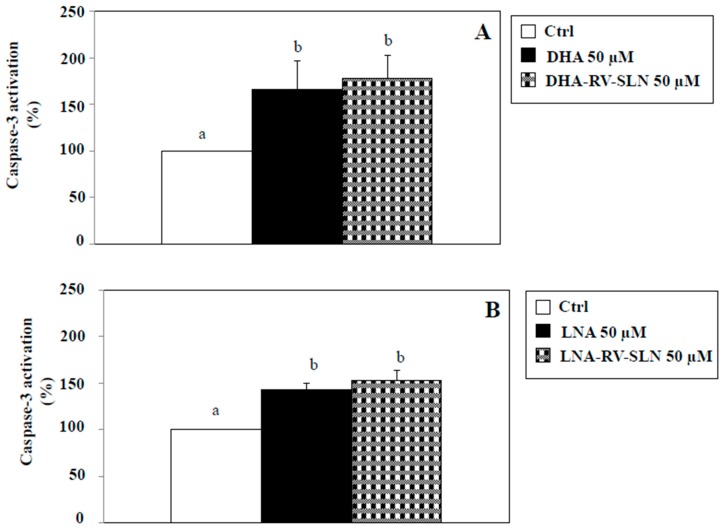
Effect of free LNA or LNA encapsulated in RV-SLN on caspase-3 activation in HT-29 CRC cells. (**A**) Effect of a 48 h-treatment with 50 µM DHA or (**B**) LNA in their free form or encapsulated in RV-SLN on caspase-3 activation in HT-29 human CRC cells. Data are the means ± SD of three different experiments. Values not sharing the same superscript are significantly different (*p* < 0.05, One-way ANOVA followed by Tukey’s test).

We next evaluated if in our experimental model DHA and LNA, either in the free or encapsulated form, were able to modify apoptosis. Apoptosis induction is one of the main mechanisms proposed to explain the cancer cell growth-inhibitory effects omega-3 PUFA [16,36,37]. We evaluated the activation of caspase-3, known to be one of the main executioner caspases involved in the apoptotic process [38]. Figure 6 shows the effect of a 48 h-treatment with DHA (Figure 6A) and LNA (Figure 6B) at the concentration of 50 µM, either in their free or RV-SLN encapsulated form. We used this concentration since it was the most efficacious in inhibiting CRC cell growth (Figure 4 and Figure 5). We found that the two FA, both in the free or encapsulated form were able to significantly (*p* < 0.05) induce caspase-3 activation (induction vs. control, 50 µM DHA: 65.6%; 50 µM LNA, 43.1%; 50 µM DHA-RV-SLN, 78%; 50 µM LNA-RV-SLN, 52.9%). It should be noticed that a slight, even though not significant, further increase in caspase-3 activation was observed when both DHA and LNA were encapsulated in RV-SLN as compared to their free forms. However, these slight increases could not explain the enhanced efficiency in inhibiting HT-29 cancer cell growth showed by omega-3 when encapsulated into RV-SLN.

### 2.6. Effects of RV-SLN on Cell Proliferation

**Figure 7:**
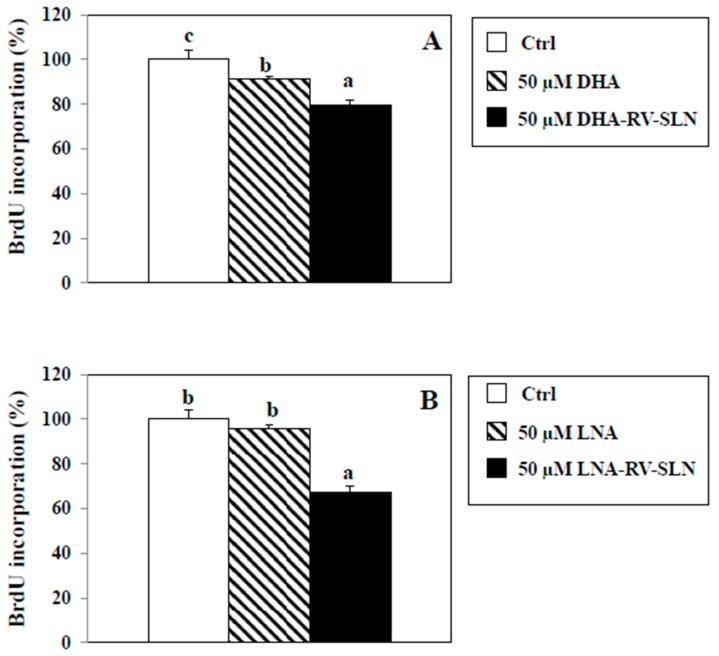
Effect of free LNA or LNA encapsulated in RV-SLN on HT-29 CRC cell proliferation. (**A**) Effect of a 48 h-treatment with 50 µM DHA or (**B**) LNA in their free or encapsulated form on BrdU incorporation in HT-29 human CRC cells. Data are the means ± SD of three different experiments. Values not sharing the same superscript are significantly different (*p* < 0.05, One-way ANOVA followed by Tukey’s test).

As an alternative mechanism, we hypothesized that DHA and LNA could modulate HT-29 cancer cell proliferation depending on how they are administered (i.e., as free FA or encapsulated in RV-SLN). To this aim, we evaluated HT-29 cancer cell proliferation by measuring the pyrimidine analog 5-bromo-2-deoxyuridine (BrdU) incorporation into DNA, in the presence of DHA or LNA, administered either in the free or encapsulated form (Figure 7). We found that DHA, in its free form, was able to slightly, but significantly inhibit cell proliferation as compared to control (9% inhibition, *p* < 0.05), and that DHA encapsulated in SLN induced a significantly higher inhibition of cell proliferation (DHA-RV-SLN inhibition vs. control: 20.4%, *p* < 0.01; DHA-RV-SLN inhibition vs. free DHA: 11.6%, *p* < 0.01) (Figure 7A). It is noteworthy that LNA, which in its free form showed only a non-significant tendency to inhibit HT-29 cell proliferation, after encapsulation enhanced markedly and significantly inhibited cell proliferation (LNA-RV-SLN inhibition vs. control: 57.3%, *p* < 0.001) (Figure 7B).

## 3. Discussion

The aim of this work was to increase the delivery efficiency of the omega-3 PUFA LNA and DHA to CRC cells, as well as to enhance their antineoplastic activity. Thus, we designed and prepared by the microemulsion technique a new type of SLN containing in their matrix stearic acid esterified to RV, and used them for the encapsulation of either LNA or DHA. We hypothesized that the use of natural and edible components for the encapsulation of omega-3 could be a particularly interesting strategy to offer protection to these highly unstable nutraceuticals and improve their activity. We chose stearic acid and RV as possible components of the matrix of the nanoparticles for a series of reasons. Stearic acid was used to increase the hydrophobicity of the omega-3 PUFA and potentially increase the efficiency of their delivery. It was selected among other possible components showing this property also for its safety, since it is known that it is able to lower LDL cholesterol compared with other saturated FA [39]. In agreement, stearic acid-containing SLN were recently used to optimize the delivery of a series of other compounds (vitamin D3, atorvastatin, dimethyl fumarate) [40,41,42]. Moreover, considering the positive associations recently found between high levels of omega-3 PUFA intake and prostate cancer [25], it appeared also very interesting the observation that stearic acid could protect prostate from carcinogenesis [43,44]. RV was used as a component of the SLN matrix with the intent to reduce the oxidative degradation that may dramatically decrease both the bioavailability and the antineoplastic activity of the administered omega-3 PUFA. RV was selected among others showing comparable powerful antioxidant properties, since, similarly to omega-3 PUFA, plenty of results have shown their “multitargeted” mode of action in CRC prevention [28]. Several pathways/targets involved in CRC carcinogenesis have been reported to be regulated in an antineoplastic sense by both RV and omega-3 PUFA [17,45,46], and both of them were shown to act as inducers of apoptosis and inhibitors of pro-survival factors [17,18,47,48,49,50]. Finally, both the nutraceuticals have also been reported to chemosensitize these cancer cells to 5-fluorouracil [19,51,52,53] a conventional antineoplastic drug widely used in CRC therapy.

Both the sphere-shaped nanoparticles designed and constructed by us, and containing DHA or LNA (DHA-RV-SLN, LNA-RV-SLN), resulted to be quite homogenous for dimensions, and showed high values of encapsulation efficiency, suggestive of a high chemical affinity of the two omega-3 PUFA for the ester composing the RV-SLN. In addition, when tested in the presence of microsomal membranes challenged by a lipophilic pro-oxidant (*t*-BOOH), the RV-SLN were able to significantly protect the membranes from lipoperoxidation. All these characteristics suggested that the nanoparticles could be particularly apt to carry the two omega-3 PUFA to CRC cells. As a matter of fact, when the DHA- or LNA-SLN were administered in vitro to HT-29 CRC cells the efficiency of incorporation of both the FA into HT-29 cells resulted to be enhanced markedly and significantly, as demonstrated by the significant increase in their respective cellular levels. Moreover, the encapsulation of LNA in RV-SLN induced a significant and a more conspicuous cellular increase also in both its metabolic products, EPA and DHA, known to be more bioactive than their precursor LNA [54,55]. In addition, only after the encapsulation, DHA was efficiently retroconverted into EPA. The increased efficiency of cancer cells to incorporate EPA after administration of the SLN containing either its precursor LNA or its metabolic product DHA seems to be of great interest, since several reports have demonstrated the ability of EPA or its derivatives to exert a protective action in animals and patients at high risk for CRC [56,57,58,59]. Particularly, the possibility of efficiently increasing (by almost three folds) the content of EPA in colonic cells by administering LNA-RV-SLN seems particularly noteworthy, since the direct intake of EPA from fish or after its purification is much less sustainable and affordable than the use of LNA for encapsulation, being this metabolic precursor of EPA largely found in vegetables and seeds.

Moreover, we observed also that the encapsulation of both DHA and LNA in RV-SLN made them more efficient in inhibiting HT-29 CRC cell growth, if compared to DHA or LNA given as free FA. These effects were confirmed also in a different human CRC cell line (HCT116) with comparable results. However, in these cells DHA-RV-SLN or LNA-RV-SLN showed significant increased cell growth inhibition as compared to free DHA or LNA only at the highest concentrations tested (DHA: at 50 µM; LNA: at 10 and 50 µM). Moreover, we found that the higher efficiency of encapsulated PUFA in inhibiting CRC cells growth was more related to their ability to better suppress cell proliferation than induce apoptosis at a greater extent. This could be explained by the fact that both the free FA were already able *per se* to markedly and significantly induce apoptosis in these CRC cells. The observation that free DHA can exert a considerable pro-apoptotic effect in CRC cells was in agreement with plenty of findings obtained previously by both us and others [20,36,60,61]. Instead, the studies reporting a pro-apoptotic effect of free LNA either in CRC cells [61,62], or in other kinds of cancer cells [63,64,65,66] are less numerous, and generally performed using concentrations of free LNA higher (60–120 µM) than those found able to significantly induce apoptosis of CRC cells in the present study. Interestingly, it was also previously reported [62] that concentrations of free LNA much higher than those used here by us (550–1000 µM vs. 50 µM) were able to inhibit the proliferation of the CRC CaCo2 cell line. In keeping, more recently, the inhibition of cell proliferation and invasive potential of human (HT-29 and HCT116) and murine (MCA38) CRC cells was also reported by using even higher concentrations of free LNA (1–5 mM), that are not achievable in human serum even following extremely high levels of LNA dietary supplementations [67]. On these bases, it seems of great interest that, even though we did not observe any inhibitory effect on cell proliferation by using free LNA at a much lower concentration (50 µM), we found that its encapsulation in RV-SLN, made this FA able to markedly and significantly inhibit CRC cell proliferation. Similarly, we demonstrated that the encapsulation of DHA in RV-SLN further enhanced the slight-even though significant-inhibitory effect exhibited by free DHA on CRC cell proliferation.

It should be underlined that the higher antineoplastic efficiency of LNA encapsulated in RV-SLN is in agreement with our previous finding [31] according to which its encapsulation in a lipid matrix containing a liposoluble antioxidant (α-tocopherol) increased its antineoplastic activity towards melanoma cells growing in vitro. Moreover, our finding corroborates the hypothesis that nanoparticles containing antioxidant factors may offer protection to highly peroxidable PUFA against degradation [31], and intensify their antineoplastic activity. Actually, all these results seem to be contradicted by the recent finding of Roy et al. [68] who observed that DHA encapsulated in polymeric nanoparticles constituted by poly-ε-caprolactone was less potent than non-encapsulated DHA, but that its ability to inhibit cell growth was restored by its co-encapsulation with a pro-oxidant such as H_2_O_2_, and completely suppressed by the co-presence of vitamin E in the nanoparticles. However, this experimental model is completely different from the one used by us (breast cancer cells vs. CRC cancer cells, and different composition of the matrix encapsulating DHA). Moreover, it seems plausible that RV, as one of the two lipid matrix constituents in our SLN, and a known multitargeted antineoplastic agent, besides potentially acting as an antioxidant to prevent DHA peroxidation, could also affect, either separately from DHA or in synergism with it, a variety of targets/pathways related to cancer cell growth. This pleiotropic action of RV could explain the potentiation in the DHA inhibitory effect on CRC cancer.

## 4. Materials and Methods

### 4.1. Reagents

Butanol, chloroform, isopropanol, methanol, *n*-hexane and tetrahydrofuran (THF) were purchased from Carlo Erba Reagents (Milan, Italy). Alpha-linolenic acid (LNA), docosahexaenoic acid (DHA), Resveratrol, stearic acid, polyoxyethylene (20) sorbitan monooleate (Tween-20), taurodeoxycolic acid, 4-dimethylaminopyridine (DMAP), dicyclohexylcarbodiimide DCC), sodium taurocholate hydrate, trichloroacetic acid (TCA), thiobarbituric acid (TBA), butylated hydroxytoluene (BHT), deuterated chloroform (CDCl_3_) were purchased from Sigma-Aldrich (Sigma Chemical Co., St. Louis, MO, USA).

### 4.2. Instruments

A Jasco 4200 using KBr disks was used to measure the FT-IR spectra and a Bruker VM-300 ACP was used to acquire the ^1^H-MNR spectra. The chemical shifts referred to the solvent were expressed in δ. A JASCO UV-530 equipment was used to analyze the UV–Vis spectra. To obtain the SLN a Silverson homogenizer (Model SL2, Chesham Bucks, UK) was used, and an Amicon TCF2A ultrafiltration system was used to wash the product (Amicon Grace, Beverley, MA USA; membrane Amicon Diaflo YM 100). Sample lyophilization was performed by using a “Freezing-drying” Micro Modulyo (Edwards, Tewksbury, MA, USA) equipment. The analyses of nanoparticle dimensions were carried out through a dynamic laser light scattering Brookhaven 90 Plus (Holtsville, NY, USA) particle size analyzer (PSA) and scanning electron microscopy (SEM) by using a JEOL JSMT 300 A microscope (JSMT, Jeol Ltd., Tokyo, Japan).

### 4.3. Synthesis of Resveratrol Stearate

In a three-necked flask, equipped with condenser, dropping funnel and magnetic stirrer, accurately flamed and maintained under an inert atmosphere, were placed 20 mL of dry chloroform to which are added 1.25 g of stearic acid (4.4 × 10^−3^ mol), 0.907 g of DCC (4.4 × 10^−3^ mol) and 5.4 × 10^−3^ g of DMAP (4.4 × 10^−5^ mol). The solution was kept under stirring for 10 min at room temperature until complete dissolution of DMAP and DCC. Next, 1 g of Resveratrol (4.4 × 10^−3^ mol) dissolved in dry chloroform was slowly added. The reaction was left under stirring at room temperature for 24 h and monitored by TLC (Thin Layer Chromatography) on silica plates using as eluent mixture CHCl_3_: methanol in a ratio 9:1. At the end it was obtained a solution with a precipitate that was removed by filtration. After that, the filtrate was completely stripped of the solvent at reduced pressure. It was obtained a white residue which was purified by chromatography column (eluent mixture CHCl_3_:methanol, 9.5:0.5) and characterized by FT-IR spectroscopy and ^1^H-NMR.

### 4.4. SLN Preparation and Characterization

SLN were prepared by a microemulsion technique at moderate temperature according to Gasco procedure [69] as shown in Table 2. Briefly, Resveratrol-stearate in the absence and/or in the presence of the omega-3 PUFA (DHA or LNA) were melted at 60–65 °C. A warm water solution of sodium taurocholate, butanol and Tween-80 was then added to obtain an optically transparent system. The warm microemulsion was immediately dispersed in cold water (~2 °C) under high-speed homogenation (Model SL2, Silverson Machines Ltd., Buckinghamshire, UK) at 8000 rpm for 15 min. The volume ratio of warm microemulsion to cold water was 1:20. Dispersions were washed two times using an Amicon TCF2A ultrafiltration system (AmiconGrace, Beverley, MA, USA; Amicon Diaflo membrane YM 100).

Particles’ size was determined by scanning electron microscopy (SEM) and dynamic light scattering (DLLS) using a 90 Plus PSA (Brookhaven Instruments Corporation, Holtsville, NY, USA) at 25 °C by measuring the autocorrelation function at 90° scattering angle. Cells were filled with 100 μL of sample solution and diluted to 4 mL with filtered (0.22 μm) water. The polydispersity index (PI) indicating the measure of the nanoparticle population distribution [70] was also determined. Six separate measurements were made to derive the average. Data were fitted by the method of inverse “Laplace transformation” and Contin [71,72].

### 4.5. Percentage of Entrapped DHA or LNA into RV-SLN

The encapsulation efficiency of SLN formulations was evaluated through a spectrophotometer UV-Vis Jasco V-530. Briefly, the amount of unencapsulated DHA or LNA in the SLN was removed by centrifugation (at 8000 rpm for 30 min) and filtration. Subsequently the obtained samples were diluted in methanol (1:9) and analyzed. The absorbance (Å) of the samples was measured using quartz cells with a thickness of 1 cm and operating at specific wavelengths for each compound (λ_(DHA)_ = 233 nm; λ_(LNA)_ = 268 nm). The encapsulation efficiency (EE%) represents the percentage of active substance encapsulated in SLN expressed referring to the initial substance amount used. This is calculated using the Equation (1):

(1) $$EE\%=\frac{g_{f}}{g_{i}}\times 100$$

where *g_i_* indicates the grams of substance initially used and *g_f_* the final amount of substance effectively entrapped into nanoparticles.

### 4.6. Evaluation of Nanoparticle Antioxidant Activity

Antioxidant ability of SLN was evaluated by the assay of MDA, which is a marker of lipid peroxidation. Rat liver microsomal membranes were prepared following the procedure previously described [31,32,33,34,73] and incubated in the presence or in the absence of RV-SLN. An aliquot (0.8 μM stearate resveratrol) of RV-SLN was added to 1 mL of microsomal membranes (0.5 mg of proteins), and subsequently *tert*-butylhydroperoxide (*t*-BOOH) was also added at a final concentration of 0.25 mM. The microsomal suspension was then incubated at 37 °C under stirring, in the dark for one hour. Subsequently, 1 mL of each sample was added to a solution consisting of 3.0 mL of trichloroacetic acid (TCA) 0.5%, 0.5 mL of thiobarbituric acid (TBA) (TBA 0.4% in HCl 0.2 M and distilled H_2_O, 2:1 *v*/*v*) and 0.07 mL of butylated hydroxytoluene (BHT) 0.2% in 95% ethanol. The samples were then incubated in a bath at 90 °C for 45 min and then centrifuged. The production of MDA was detected spectrophotometrically at 535 nm. The experiment was repeated in triplicate.

### 4.7. Lipid Extraction and Fatty Acid Analysis

Cell lipids were extracted according to Folch with minor modifications [74]. Briefly, cell pellets were homogenized with chloroform/methanol 1:2, centrifuged to recover the lipid extract and extracted again two times with chloroform/methanol, 2:1 and 1:1 (*v*/*v*) respectively. Each solvent used for extraction and analysis contained 0.045 mM 3,5-di-tert-4-butylhydroxytoluene (BHT) to avoid PUFAs oxidation.

The fatty acid composition was determined by gas chromatography (Agilent Technologies 6850 series II, Santa Clara, CA, USA) as previously described [75]. Lipids were derivatized (sodium methoxide in methanol 3.33% (*w*/*v*)) to obtain fatty acid methyl esters (FAME). Prior to derivatization, a known amount of internal standard (C17:0 triglyceride) was added to each sample to correct for yield and recovery of the reaction. A standard mixture containing all fatty acid methylesters (Sigma Aldrich) was injected as calibration for quantitative analysis.

### 4.8. Cell Lines and Treatments

HT-29 and HCT116 human CRC cells were purchased by American Type Culture Collection (ATCC, Manassas, VA, USA). HT-29 cells were cultured in RPMI 1640 medium containing glutamine (2 mM), antibiotics (100 µg/mL Streptomycin and 100 U/mL Penicillin) in the presence of 10% foetal bovine serum (FBS). HCT116 cells were grown in DMEM medium containing glutamine (2 mM), antibiotics (100 µg/mL Streptomycin and 100 U/mL Penicillin), non-essential aminoacids, sodium pyruvate and 10% FBS. Cells were incubated at 37 °C in a humified atmosphere containing 5% CO_2_ and maintained in an exponential growth phase by trypsinization and seeding at the concentration of 3 × 10^5^ cells/mL twice a week. The cells were treated with increasing concentrations (5–50 µM) of free LNA or DHA from 10 mM stock solutions in ethanol. In this case, control cells were treated with equal amounts of vehicle alone (never exceeding 0.05%, *v*/*v*) in culture medium.

For the experiments performed using the RV-SLN as a vehicle for LNA and DHA delivery to cells, stock solutions of empty RV-SLN and RV-SLN charged with LNA or DHA were prepared in the culture medium. Different amounts of these stock solutions, calculated on the basis of the encapsulation efficiency (see Section 3.5) of DHA and LNA in the RV-SLN, were administered to the cells in order to obtain the same concentration used for LNA and DHA in the free form (5–50 µM). In this case, control cells were treated with the same amount of empty RV-SLN stock solutions.

### 4.9. Cell Growth Evaluation

HT-29 and HCT116 CRC cells were seeded in 24-well multiwell culture plates at the concentration of 3 × 10^4^ cell/mL. After 24 h, cell culture medium was removed and replaced with fresh culture medium containing or not DHA or LNA in ethanol (5, 10 and 50 µM) or containing DHA-RV SLN or LNA-RV-SLN at the same concentrations. At the indicated time points (24, 48 and 72 h), cells were washed with phosphate buffered saline (PBS, pH 7.4), trypsinized, centrifuged and counted by using a haemocytometer Neubauer chamber. Cell viability was evaluated by the Trypan blue dye exclusion method [76].

### 4.10. Analysis of Apoptosis

Apoptosis evaluation was performed by using a colorimetric commercial kit which allows to detect the activation of caspase-3, the main executioner caspase in apoptotic process [38], through the spectrophotometric detection of *p*-nitroaniline chromophore (*p*NA) following the enzymatic cleavage of the substrate DEVD-*p*NA (Caspase-3/CPP32 Colorimetric Assay Kit, Enzo Life Sciences, NY, USA). Briefly, HT-29 cells were seeded at the concentration of 3 × 10^5^ cells/mL, and, after 24 h, the culture medium was removed and replaced with fresh culture medium containing or not free DHA or LNA solubilized in ethanol at the concentration of 50 µM or containing DHA-RV-SLN or LNA-RV-SLN at the same concentration. At the indicated time point (48 h), the cells were washed with ice cold PBS (pH 7.4), trypsinized, centrifuged, resuspended in PBS and counted. 50 µL of cold lysis buffer were added to 1.5 × 10^5^ cells, samples were incubated on ice for 10 min and then they were centrifuged at 13,000 rpm for 1 min. Surnatants were collected and protein concentration was evaluated through the Bradford method [77]. For each sample, 200 µg proteins were diluted in 50 µL of reaction buffer containing 10 mM dithiotreitol (DTT) and 200 µM DEVD-*p*NA. Samples were incubated at 37 °C for 2 h and then *p*NA light emission was quantitated by spectrophotometrical detection at a wavelength of 405 nm.

### 4.11. Cell Proliferation Analysis

The percentage of proliferating cells was evaluated by the use of the 5-Bromo-2-deoxyuridine (5-BrdU) Cell Proliferation Assay Kit (BioVision, Milpitas, CA, USA) which allows to detect proliferating cells by measuring the incorporation of BrdU, a pyrimidine analog, in the newly synthetized DNA in place of thymidine. The analysis was performed following the manufacturer’s instructions. Briefly, HT-29 cells were seeded at the concentration of 10^4^ cells/well in a 96-well Multiwell plate. After 24 h, culture medium was removed and replaced with fresh culture medium containing or not free DHA or LNA solubilized in ethanol at the concentration of 50 µM or containing DHA-RV-SLN or LNA-RV-SLN at the same concentration. After 48 h treatment, the cells were incubated in the presence of BrdU at 37 °C for further 4 h. Then culture medium was removed and 100 µL of Fixing/Denaturing solution was added to each well. Samples were incubated at room temperature for 30 min. After removing the Fixing/Denaturing solution, 100 µL of BrdU Detection Antibody solution were added. After 1 h of incubation at 37 °C with gentle shaking, wells were washed twice with Washing Solution. Anti-mouse HRP-linked Antibody Solution (100 µL) was then added to the wells. The solution was removed, wells were washed three times and then 100 µL of TMB substrate were added. A stop solution was added after 30 min and color development was measured at 450 nm wavelength.

### 4.12. Statistical Analysis

All data are presented as means ±SD for three separate experiments. Data were analyzed by two-way ANOVA test, followed by Bonferroni’s post-test, using the GraphPAD Prism4 software (GraphPad Software, La Jolla, CA, USA) in Figure 2, by the unpaired *t*-test in Figure 4 and Figure 5, and through One-Way Analysis of Variance (ANOVA) followed by Tukey’s test in Figure 3, Figure 6 and Figure 7. Differences were considered statistically significant at *p* < 0.05.

## 5. Conclusions

Overall, the results of our investigation conducted in vitro on HT-29 CRC cells demonstrated that DHA and LNA were better incorporated in the cells, and showed a markedly enhanced antineoplastic activity when administered encapsulated in the SLN constructed by us, and containing in their matrix stearic acid and the antioxidant and antineoplastic agent RV. The remarkable and enhanced increase in cellular DHA and EPA content after cell incubation with their precursor LNA encapsulated in the SLN appears particularly interesting. As a matter of fact, through a quite cheap and ecologically sustainable administration of LNA in its encapsulated form, it could be possible to considerably enrich colonic cells not only with LNA itself, but also with both its metabolic products EPA and DHA, known to be more bioactive than LNA as antineoplastic agents in CRC. These results suggest that these formulations could be optimal candidates for further in vivo studies for the development of innovative treatment of CRC.

## Figures and Tables

**Table 1:** Average diameter and polydispersity index of SLN.

Formulation	Mean Particle Size (nm)	Polydispersity Index (PI)
RV-SLN	571 ± 6	0.198 ± 0.023
LNA-RV-SLN	842.2 ± 1.3	0.126 ± 0.017
DHA-RV-SLN	1000 ± 1.8	0.220 ± 0.020

RV-SLN: resveratrol-based solid lipid nanoparticles; LNA-RV-SLN: linolenic acid-loaded resveratrol-based solid lipid nanoparticles; DHA-RV-SLN: docosahexaenoic-loaded solid lipid nanoparticles.

**Table 2:** Ingredients of DHA- and LNA-RV-SLN.

Resveratrol Stearate (g)	Tween 80 (mL)	1-Butanol (mL)	Biliary Salt (g)	Water (mL)	DHA (g)	LNA (g)
0.1	0.62	0.026	0.036	0.29	6.1 × 10^−3^	5.2 × 10^−3^
